# High Levels of Treatment Success and Zero Relapse in Multidrug-Resistant Tuberculosis Patients Receiving a Levofloxacin-Based Shorter Treatment Regimen in Vietnam

**DOI:** 10.3390/tropicalmed5010043

**Published:** 2020-03-10

**Authors:** Le T. N. Anh, Ajay M. V. Kumar, Gomathi Ramaswamy, Thurain Htun, Thuy Thanh Hoang Thi, Giang Hoai Nguyen, Mamel Quelapio, Agnes Gebhard, Hoa Binh Nguyen, Nhung Viet Nguyen

**Affiliations:** 1 Vietnam Integrated Center for TB and Respirology Research, National Lung Hospital, Ha Noi 100000, Vietnam; nguyenbinhhoatb@yahoo.com (H.B.N.); vietnhung@yahoo.com (N.V.N.); 2 International Union Against Tuberculosis and Lung Disease, South East Asia Office, New Delhi 110016, India; akumar@theunion.org; 3 International Union Against Tuberculosis and Lung Disease, 75006 Paris, France; 4 Yenepoya Medical College, Yenepoya (Deemed to be University), Mangaluru 575018, India; 5 National Centre of Excellence and Advanced Research on Anemia Control, Centre for Community Medicine, All India Institute of Medical Sciences, New Delhi 110029, India; gmthramaswamy@gmail.com; 6 International Union Against Tuberculosis and Lung Disease, Mandalay 05021, Myanmar; drthurain07@gmail.com; 7 Programmatic Management of Drug Resistant Tuberculosis Unit, National Lung Hospital, Ha Noi 100000, Vietnam; hoangthanht@gmail.com; 8 Interactive Research and Development, Ho Chi Minh 700000, Vietnam; nhgiang@gmail.com; 9 KNCV Tuberculosis Foundation, 2596 BC The Hague, The Netherlands; mameldquelapio@gmail.com (M.Q.); agnes.gebhard@kncvtbc.org (A.G.)

**Keywords:** STR, Bangladesh regimen, operational research, SORT IT

## Abstract

Vietnam has been using a levofloxacin-based shorter treatment regimen (STR) for rifampicin resistant/multidrug-resistant tuberculosis (RR/MDR-TB) patients since 2016 on a pilot basis. This regimen lasts for 9–11 months and is provided to RR/MDR-TB patients without second-line drug resistance. We report the treatment outcomes and factors associated with unsuccessful outcomes. We conducted a cohort study involving secondary analysis of data extracted from electronic patient records maintained by the national TB program (NTP). Of the 302 patients enrolled from April 2016 to June 2018, 259 (85.8%) patients were successfully treated (246 cured and 13 ‘treatment completed’). Unsuccessful outcomes included: treatment failure (16, 5.3%), loss to follow-up (14, 4.6%) and death (13, 4.3%). HIV-positive TB patients, those aged ≥65 years and patients culture-positive at baseline had a higher risk of unsuccessful outcomes. In a sub-group of patients enrolled in 2016 (n = 99) and assessed at 12 months after treatment completion, no cases of relapse were identified. These findings vindicate the decision of the Vietnam NTP to use a levofloxacin-based STR in RR/MDR-TB patients without second-line drug resistance. This regimen may be considered for nationwide scale-up after a detailed assessment of adverse drug events.

## 1. Introduction

Multidrug-resistant tuberculosis (MDR-TB), defined as TB resistant to both isoniazid and rifampicin, is a global public health challenge. In 2018, an estimated 484,000 people developed TB that was resistant to rifampicin (RR-TB), and of these, 78% had MDR-TB [1]. Treatment coverage and treatment success rates among MDR-TB patients have been poor. Globally, only 32% of estimated RR/MDR-TB patients were treated in 2018, and only 56% of them were successfully treated [1], mostly because of challenges related to the long duration of the treatment and toxic drugs. 

There has been great progress in the development and availability of new diagnostic tools, new drugs and new treatment regimens for MDR-TB. The new diagnostic tools include Xpert^®^ MTB/RIF assay (including the Ultra [2] and Omni versions [3] and a new assay under development to diagnose resistance to second-line drugs [4]) and line probe assay (LPA) to diagnose resistance to the most important first-line and second-line drugs (SLD) [5]. On the treatment front, there are two new drugs in the armamentarium against TB—Bedaquiline and Delamanid—and some of the existing drugs are being repurposed for use in MDR-TB treatment [6,7,8]. Further, the duration of treatment has been shortened. Owing to a large body of evidence accumulated over the past few years, WHO recommends use of standardized shorter treatment regimen (STR) for patients with MDR-TB (in whom SLD resistance has been excluded) [9,10,11,12,13,14,15]. 

There has been a debate about the choice of fluoroquinolone to be used in the STRs. While the initial studies in Bangladesh, Niger and Cameron used Gatifloxacin [10,11,12], later studies in nine African countries and the STREAM trial used Moxifloxacin [14,16], owing to concerns about dysglycaemia (low or high blood sugar) caused by Gatifloxacin and the subsequent withdrawal of the drug from the market [17]. Experts have argued that these concerns about safety of Gatifloxacin have to be balanced against those of Moxifloxacin [18,19], which is not immune to causing dysglycemia [20]. In addition, Moxifloxacin causes QT interval prolongation (a measure of delayed ventricular repolarization as measured on electrocardiogram) leading to cardiac arrhythmias and sudden death [21]. 

Owing to these concerns, there has been interest in the use of Levofloxacin in STRs [22] because it does not significantly prolong the mean QT interval [23] and the initial data support its effectiveness [24]. There has been limited evidence about the effectiveness of Levofloxacin-based STRs among MDR-TB patients, barring a sub-group analysis from Bangladesh showing high treatment success [25]. Vietnam is one of the unique settings, which has always used a Levofloxacin-based STR. A systematic assessment of the treatment outcomes in Vietnam has the potential to contribute to the global evidence base on this issue.

So, we conducted an operational research with the following objectives: (i) to describe the demographic and clinical profile of MDR-TB patients who received the Levofloxacin-based STR from April 2016 to June 2018 under the national tuberculosis program (NTP) of Vietnam (ii) to assess their treatment outcomes, including 12 months’ recurrence in a sub-group of patients and (iii) to determine the factors associated with unsuccessful treatment outcomes.

## 2. Materials and Methods

### 2.1. Study Design

This was a cohort study involving secondary analysis of routinely collected program data.

### 2.2. Setting

Vietnam is a lower-middle income country situated in East Asia with a population of 92 million. The country has 63 provinces, 673 districts and 10,925 communes. Vietnam is considered as one of the 30 high-burden countries in the world, with a MDR-TB prevalence of 3.6% and 17% among new and previously treated TB patients respectively [1]. Vietnam started the programmatic management of drug resistant TB (PMDT) in 2009 using the conventional regimen (20–24 months), which was scaled-up nationwide in 2016. The latest available outcome data indicates a relatively high treatment success of 68% among a cohort of MDR-TB patients enrolled in 2016, which is substantially higher than the global average [1].

The NTP network follows the hierarchy of the health system. The National Lung Hospital in Hanoi is the national referral hospital. There are three sub-national TB referral hospitals: Pham Ngoc Thach hospital in Ho Chi Minh City, TB and Lung Hospital in Da Nang Province and TB and Lung hospital in Can Tho province. There are 63 provincial TB hospitals or TB units as part of the provincial preventive medicine department. Each of the districts has a TB coordinator and each commune has a commune health worker responsible for TB. The commune health workers educate the community on TB, refer people with symptoms suggestive for TB and provide treatment support.

#### 2.2.1. Diagnosis and Treatment of MDR-TB

Under the PMDT program, all TB patients are screened for rifampicin resistance using Xpert^®^ MTB/RIF assay. Patients diagnosed with rifampicin resistance are then tested for evidence of second-line drug resistance (fluoroquinolone, second-line injectables) using LPA (Genotype^®^ MTBDRsl assay, Hain Lifescience, Nehren, Germany) and/or phenotypic culture (either in liquid media using BACTEC MGIT 960 instrument or solid Lowenstein–Jensen media) and drug susceptibility test (DST using proportion method) at the sub-national reference laboratories. All the laboratories are quality-assured and under the supervision of supranational reference laboratory at Adelaide, Australia.

While waiting for the results of SLD resistance, patients are referred to the provincial hospital for conducting baseline tests which include testing for HIV, diabetes, hepatic and renal function, audiometry and routine biochemistry and assessing if they are eligible for STR. Accordingly, a decision is made by a committee to start STR. All the patients are hospitalized for a period of two to four weeks at the time of starting treatment. This is followed by ambulatory treatment delivered under direct observation of the health workers at either district or commune levels. If the results of the initial LPA or DST show that there is resistance to SLDs, the treatment is stopped and the patient is shifted to an individualized regimen based on the drug resistance pattern. 

For all patients, a family (or friend) is designated as treatment supporter whose role is to remind the patient to consume the medicines regularly and visit the health facility for follow-up. Psychosocial support is provided by the community-based organizations (Women’s Union, Farmers’ Union, Youth Union, and the Red Cross) which run the ‘TB patient clubs’. In addition, food allowance, hospitalization costs during intensive phase and transport costs incurred during follow-up visits are reimbursed by the NTP.

#### 2.2.2. The Treatment Regimen

The STR is provided for 9–11 months and consists of two phases: intensive phase for 4–6 months and continuation phase for a fixed duration of 5 months (Table 1). The intensive phase consists of the following drugs: Levofloxacin, Kanamycin, Clofazimine, Prothionamide, Ethambutol, high-dose Isoniazid, and Pyrazinamide, given daily. The continuation phase consists of Levofloxacin, Clofazimine, Ethambutol and Pyrazinamide, given daily. 

#### 2.2.3. Follow-Up Schedule during the Treatment

The treatment response is monitored using clinical examination, follow-up sputum smear microscopy and culture, done monthly. If the sputum smear is positive at four months of treatment, the intensive phase is extended for a month or two. During the extended intensive phase, kanamycin is provided intermittently (thrice a week) to reduce toxicity. Patients are switched to continuation phase once two consecutive culture results taken at least 30 days apart in intensive phase are negative. If the person remains culture positive at the end of the 4th month or later, or reverts after conversion, DST (for second-line drugs) is done for every positive culture and the patient is shifted to an individualized regimen based on the susceptibility pattern. A treatment outcome is assigned for each person. The operational definitions of treatment outcomes and other outcome indicators are detailed in Table 2 [26].

Patients who experience adverse drug events are followed-up until clinical recovery is complete and laboratory results have returned to normal, or until the event has stabilized. All the treatment related details are recorded in the TB treatment card by the health care providers and electronically captured in the e-TB manager system as per national guidelines. 

#### 2.2.4. Follow-Up Schedule Post-Treatment

Patient whose treatment outcome is either cured or completed are invited to come back to the provincial hospital for post-treatment follow-up every six months till 24 months after treatment completion. TB symptom screening and chest radiography are done at each follow-up visit. If the patient has TB symptoms (cough ≥ 2 weeks, hemoptysis) or abnormalities on chest X-ray, s/he is asked to produce a sputum sample which is tested using Xpert MTB/RIF and culture and drug susceptibility test. If patient does not return for follow-up as scheduled, health staffs at the provincial hospital follow-up over phone or conduct a home visit for screening.

### 2.3. Study Population

All the RR/MDR-TB patients who received the levofloxacin-based STR under NTP in Vietnam between April 2016 and June 2018 were included in the study. All the patients had a confirmed rifampicin resistance result by a genotypic (Xpert MTB/RIF assay) or a phenotypic test (culture and DST). STR was implemented on a pilot basis in the seven sites of the country which include Ha Noi, Ho Chi Minh, Can Tho, Da Nang, Nam Dinh, Soc Trang and Khanh Hoa. The number of patients enrolled depended on the number of treatment courses available with the NTP Vietnam. Patients with known resistance to second-line drugs, children aged <15 years, pregnant or breastfeeding women, those who received SLDs for more than a month, patients with hypersensitivity to any of the drugs used were excluded from receiving the STR.

### 2.4. Data Variables, Sources of Data and Data Collection

The data were extracted from the e-TB manager and reviewed. The data of each MDR-TB patient in the e-TB system was validated by referring to the original paper-based source (treatment card) for completeness and consistency. If there was any inconsistency between data in e-TB system and treatment card, we considered the treatment card as final and updated information into the e-TB system, before extracting for final analysis.

### 2.5. Analysis and Statistics

Data was analyzed using Stata (v14.1, Statacorp, College Station, TX, USA). We described the demographic and clinical profile of patients using mean (and standard deviation), or median (and interquartile range) or frequency (and proportions) as appropriate to the type of variable and the normality of distribution. Adherence to follow-up examination was summarized using frequencies and percentages (for each month of follow-up). We initially planned to use a Cox proportional hazards model, but the proportionality assumption was not met. Hence we used a log-binomial regression to assess the factors associated with unsuccessful outcomes and calculated adjusted relative risks and 95% confidence intervals to measure associations. Since we used an exploratory approach, all the variables used in unadjusted analysis were included in multivariable model. A p value of <0.05 was considered statistically significant in all analyses.

### 2.6. Ethics Approval

Permission to conduct the study and access the data was obtained from the NTP. Ethics approval was obtained from the Scientific Committee at National Lung Hospital, Ha Noi, Vietnam (approval number 08/19) and the Ethics Advisory Group of International Union Against Tuberculosis and Lung Disease, Paris, France (approval number 16/19). As this study involved a review of existing records, the ethics committees waived the need for individual informed consent.

## 3. Results

### 3.1. Baseline Sociodemographic and Clinical Characteristics

A total of 302 MDR-TB patients received a levofloxacin-based STR during the study period. Sociodemographic and clinical characteristics are shown in Table 3. The mean (SD) age of the patients was 41.0 (14.3) years and 224 (74.2%) were men. Of the 302 patients, 117 (38.7%) were new TB cases. Sputum smear was positive among 210 (69.5%) and sputum culture was positive among 199 (65.9%) patients. About half of the patients belonged to the 33–50 kg weight band. The mean (SD) body mass index was 18.7 (2.7) kg/m2. A total of 256 (84.7%) patients were tested for HIV and of them, three (1%) were HIV-positive and only one received antiretroviral therapy (ART).

### 3.2. Adherence to Monthly Follow-Up Visits

**Figure 1:**
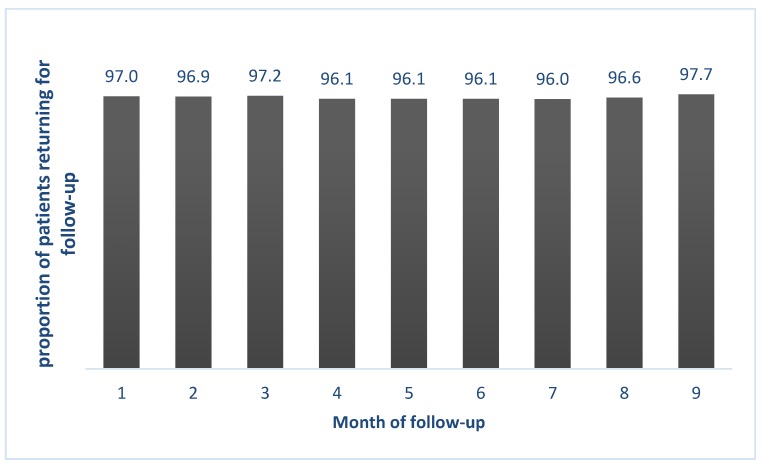
Monthly adherence to follow-up visit among MDR-TB patients who received STR from April 2016 to June 2018 in Vietnam. STR, Levofloxacin based shorter treatment regimen; MDR, Multi Drug resistance; NTP, National Tuberculosis Program.

Figure 1 depicts the extent of adherence to the follow-up visits among the study participants. The adherence rate among the study participants was more than 95% for all nine months.

### 3.3. Treatment Outcomes

Overall, 246 (81.5%) patients were cured and 13 (4.3%) completed the treatment (Table 4). Thus a total of 259 (85.8%) were successfully treated. Unsuccessful treatment outcomes such as treatment failure, loss to follow-up (LTFU) and death were observed in 16 (5.3%), 14 (4.6%) and 13 (4.3%) patients respectively. Among 275 patients with bacteriological outcome (excluding deaths and LTFU), 259 (94.2%) had a successful outcome. Patients who were culture positive at baseline had a worse outcome (due to higher failure rate) compared to those who were culture negative (Table 4). HIV-positive TB patients, those aged ≥65 years and patients with a positive baseline culture were more likely to have unsuccessful outcomes (Table 5). The median (IQR) time to death from start of treatment was 95 (51–155) days and that to LTFU was 126 (57–214) days. 

### 3.4. One-Year TB Relapse

Relapse data was available only for patients enrolled in year 2016. Among 99 patients enrolled in 2016, 79 patients had a successful treatment outcome and were contacted after 12 months of treatment completion. Everyone was screened for TB symptoms and only four patients had cough of ≥2 weeks. Everyone was advised to undergo chest X-ray, though only 51 (64.5%) patients underwent chest X-ray. A total of 43 (84.3%) patients had either abnormality on chest X-ray and/or cough ≥2 weeks. 26 out of 43 (60.5%) patients were able to produce sputum samples, which were tested using Xpert MTB/RIF assay and sputum culture. None of the patients tested had TB.

## 4. Discussion

This is the first study from Vietnam providing information on treatment outcomes of a levofloxacin-based STR among RR/MDR-TB patients managed under routine program settings. Most studies on STR have used either gatifloxacin or moxifloxacin (in normal or high doses). Levofloxacin-based STR has rarely been used and the only other patient cohort that used a levofloxacin-based STR was from Bangladesh. Thus, this study adds to the limited global evidence on the effectiveness of levofloxacin-based STR. 

One of the major limitations is that we are unable to report the information on adverse drug events in this paper. This is being assessed independently by the Vietnam Pharmacovigilance Center and will be reported in a separate paper. The other limitations include lack of information on acquired SLD resistance and relapses for the full cohort of patients. 

We found high rates of treatment success (86%) and bacteriological effectiveness (94%) in our study with no relapse reported in a sub-group. This is comparable to the results of the Bangladesh cohort, which reported a bacteriological effectiveness of 96%. We were able to obtain these results using a relatively lower dose of levofloxacin (maximum dose of 1000 mg/day), unlike the study from Bangladesh which used a high-dose levofloxacin (maximum dose of 1750 mg/day) [25]. The treatment success rates obtained using STR are also higher than the reported treatment success rates among MDR-TB patients treated with long regimen in Vietnam (68%) [1]. This comparison should though be made with caution due to possible selection bias. First, not all eligible patients received STR, because the number of patients enrolled was limited by the number of treatment courses available [27]. Second, the profile of the patients treated with STR (where SLD resistance has been ruled out) is different from those treated with the long regimen (where there may be people with unknown SLD resistance). Despite these limitations, we feel the use of STR in carefully selected RR/MDR-TB patients have the potential to improve the overall treatment success rates in Vietnam. 

The LTFU rates were low at 4.6% and this may probably be due to direct observation of treatment and several other patient-friendly initiatives which included free hospitalization, provision of food allowance and reimbursement of transport costs and use of patient clubs for improving treatment adherence. The death rates were also low at 4.3% and most of deaths occurred during the intensive phase (75%) indicating severity of illness.

Several factors were found to be associated with unsuccessful outcomes. We found that HIV-positive patients had higher risk of unfavorable outcomes. About 15% of the patients were not HIV tested and only one out of three HIV-positive TB patients received ART. This needs to be strengthened. In this era of ‘test and treat’ strategy, it is rather unacceptable to see gaps in ART uptake among HIV-infected MDR-TB patients at high risk of mortality. Also, for reasons that are unclear, people aged ≥65 years had a higher risk of unsuccessful outcome. The outcomes seem to be improving over the years with patients enrolled in 2017 and 2018 having better outcomes compared to 2016 cohort. This may be due to a higher proportion of heavily treatment-experienced chronic patients in the earlier cohort compared to the recent ones. A limitation of this analysis is that we did not have information on drug susceptibility status of other first line (like isoniazid, pyrazinamide, streptomycin) drugs at baseline, socio-economic status and education level of the patients and other confounders that may have accounted for unsuccessful outcomes. Also, the exact reasons for LTFU and death were not explored in this study. This needs to be studied in future research using qualitative research methods.

The evidence from this study adds to the debate on the choice of FLUOROQUINOLONE in the STR. A multi-country analysis published recently by Van Deun et al shows that patients treated with a Gatifloxacin-based STR (97%) had the best bacteriologic outcomes followed by Levofloxacin (96%) and Moxifloxacin (95%) [25]. The same ranking was also demonstrated in a pharmacodynamics study assessing the microbial killing capacity of fluoroquinolones [24]. Unfortunately, Gatifloxacin was withdrawn from the market in most countries in April 2006, following a study among elderly patients (mean age was 78 years and most of them had diabetes) from Canada which reported higher risk of dysglycaemia with use of Gatifloxacin [17]. These results have not been replicated since then [20]. We also feel that the results of this study cannot be applied directly to the MDR-TB patient cohorts in most settings, who are much younger and often undernourished with much less likelihood of dysglycaemia. Experience from Bangladesh and Niger shows that dysglycaemia is relatively rare (4–6%), and even when it occurs, is reversible, and can easily be diagnosed and managed under program settings [13]. In contrast, Moxifloxacin-based STRs have reported relatively lower bacteriological effectiveness (with higher rates of acquired FLUOROQUINOLONE resistance and relapses) and high incidence of QT prolongation (ranging from 11% to 15%) [15,28] and cardiac arrhythmias, which are more challenging to detect (requires an electrocardiogram) and manage in routine program conditions. This has led to calls for bringing back Gatifloxacin into the market and adding it in the WHO essential drugs list, which has generic versions available and is less expensive [18,19]. Levofloxacin is intermediate in effectiveness and seems to have a better safety profile: it is not associated with clinically significant QT prolongation (like Moxifloxacin) [23] and causes milder forms of dysglycemia when compared to Gatifloxacin. Thus, it seems levofloxacin is the next best alternative to be used in STRs, in the absence of Gatifloxacin. 

In conclusion, we found high rates of treatment success among RR/MDR-TB patients treated with a levofloxacin-based regimen in Vietnam. These results add to the global body of knowledge about effectiveness of STRs in general and specifically on the effectiveness of levofloxacin-based STRs. These findings also vindicate the decision of Vietnam NTP to use Levofloxacin as the core drug in STRs. This regimen may be considered for nationwide scale-up after a detailed assessment of adverse drug events.

## Figures and Tables

**Table 1:** Weight-based drug dosages used among MDR-TB patients treated with shorter treatment regimen in Vietnam, 2016–2018.

Drug	Months	Drug Doses by Weight Group			
		<33 kg	33–50 kg	>50–70 kg	>70 kg
Kanamycin *(Km)	1–4 (6)	0.5 g	0.75 g	0.75 g	1 g
Levofloxacin (Lx)	1–9	500 mg	750 mg	750 mg	1000 mg
Clofazimine (Cfz)	1–9	50 mg	100 mg	100 mg	100 mg
Ethambutol (E)	1–9	600 mg	800 mg	1000 mg	1200 mg
Pyrazinamide (Z)	1–9	750 mg	1500 mg	2000 mg	2000 mg
Isoniazid (H)	1–4	300 mg	400 mg	600 mg	600 mg
Prothionamide (Pto)	1–4	500 mg	500 mg	750 mg	1000 mg

* with a maximum of 0.75 g for people over 45 years of age, mg = milligram, g = gram.

**Table 2:** Treatment outcome and adherence to follow up definitions among MDR-TB patients started on shorter treatment regimen in Vietnam, 2016–2018.

Term	Definitions
Cured	Treatment completed without evidence of failure and two consecutive negative cultures taken at least 30 days apart in the continuation phase
Treatment completed	Treatment completed without evidence of failure but there is no record of two consecutive negative cultures taken at least 30 days apart in the continuation phase.
Died	A patient who dies for any reason during the course of treatment
Failure	A patient who has a positive culture after ≥6 months of treatment (except for an isolated positive culture, which is a culture preceded by ≥1 and followed ≥2 negative cultures) OR
	A patient who after an initial conversion, has a reversion after ≥6 months of treatment with two consecutive positive cultures taken at-least 30 days apart OR
	evidence of additional acquired resistance to fluoroquinolones or second-line injectables OR
	treatment terminated or need for permanent change of at least two of anti-TB drugs due to adverse drug reactions
Lost to follow-up (LTFU)	A patient whose treatment was interrupted for ≥2 consecutive months
Not evaluated	A patient for whom no treatment outcome is assigned (this includes patients “transferred out” to another treatment unit and whose treatment outcome is unknown)
Treatment success	The sum of cured and treatment completed
Unsuccessful treatment outcomes	The sum of death, lost to follow-up, failure and not evaluated
Relapse	Patient after completing a course of STR and declared “cured” or “treatment completed”, is diagnosed with another episode of confirmed RR-TB (based on Xpert MTB/RIF assay or culture) during a follow-up period of one year post-treatment
Adherence to follow-up	Number who had a follow-up smear or culture divided by number eligible for follow-up for a given month. Number eligible will be calculated by subtracting the number dead and lost to follow-up before the scheduled follow-up time.
Bacteriological effectiveness	This is calculated by dividing the number successfully treated by the number of patients who had a bacteriological outcome (excluding death, LTFU and not evaluated)

**Table 3:** Baseline sociodemographic and clinical characteristics of MDR-TB patients who received STR from April 2016 to June 2018 in Vietnam.

Characteristics		Number	(%)
Total		302	(100.0)
**Age categories in years**			
	15–24	35	(11.6)
	25–34	76	(25.2)
	35–44	77	(25.5)
	45–54	57	(18.9)
	55–64	38	(12.6)
	≥65	19	(6.2)
**Gender**			
	Male	224	(74.2)
	Female	78	(25.8)
**Weight categories**			
	<33 kg	3	(1.0)
	33–50 kg	133	(44.0)
	51–70 kg	114	(37.8)
	71–80 kg	3	(1.0)
	Missing	49	(18.2)
**Body Mass Index (kg/m^2^)**			
	Underweight (<18.5)	113	(37.4)
	Normal (18.5–22.9)	99	(32.8)
	Overweight/obese (≥23.0)	17	(5.6)
	Missing	73	(24.2)
**HIV**			
	Negative	253	(84.0)
	Positive	3	(1.0)
	Missing	46	(15.0)
**TB categories**			
	New	117	(38.7)
	Relapse	112	(37.1)
	Treatment after LTFU	2	(0.7)
	Treatment after failure	60	(19.9)
	Others	5	(1.6)
	Missing	6	(2.0)
**Sputum smear microscopy**			
	Negative	82	(27.1)
	Scanty positive	35	(11.6)
	1+ positive	77	(25.5)
	2+ positive	45	(14.9)
	3+ positive	53	(17.6)
	Unknown/missing	10	(3.3)
**Culture positive**			
	Negative	61	(20.2)
	Positive	199	(65.9)
	Unknown	42	(13.9)
**Year of enrolment**			
	2016	99	(32.8)
	2017	72	(23.8)
	2018	131	(43.4)

**Table 4:** Treatment outcomes (disaggregated by baseline culture positivity) among patients who received STR from April 2016 to June 2018 in Vietnam.

Treatment Outcomes	Culture Positive		Culture Negative		Culture Unknown		Total	
	N	(%)	N	(%)	N	(%)	N	(%)
**Total**	**199**	**(100)**	**61**	**(100)**	**42**	**(100)**	**302**	**(100)**
**Successful Outcomes**	**166**	**(83.4)**	**55**	**(90.2)**	**38**	**(90.5)**	**259**	**(85.8)**
Cured	158	(79.4)	52	(85.3)	36	(85.7)	246	(81.5)
Completed	8	(4.0)	3	(4.9)	2	(4.8)	13	(4.3)
**Unsuccessful Outcomes**	**33**	**(16.6)**	**6**	**(9.8)**	**4**	**(9.5)**	**43**	**(14.2)**
Failure	13	(6.6)	1	(1.6)	2	(4.8)	16	(5.3)
LTFU	10	(5.0)	2	(3.3)	2	(4.8)	14	(4.6)
Died	10	(5.0)	3	(4.9)	0	(0)	13	(4.3)

**Table 5:** Factors associated with unsuccessful treatment outcome among MDR-TB patients who received STR from April 2016 to June 2018 in Vietnam.

Factors		Total	Unsuccessful Outcome		RR	(95%CI)	aRR	(95%CI)
		N	n	(%)#				
Total		302	43	(14.2)				
**Age in years**								
	15–44	188	22	(11.7)	ref		ref	
	45–64	95	17	(17.9)	1.53	(0.85–2.74)	1.57	(0.84–2.93)
	≥ 65	19	4	(21.0)	1.80	(0.69–4.68)	2.97	(1.22–7.22) *
**Gender**								
	Male	224	35	(15.6)	1.52	(0.74–3.14)	1.31	(0.71–2.44)
	Female	78	8	(10.3)	ref		ref	
**BMI**								
	Under weight (<18.5)	113	15	(13.3)	1.10	(0.54–2.23)	0.98	(0.46–2.06)
	Normal (18.5–22.9)	99	12	(12.1)	ref		Ref	
	Overweight/Obese (≥23.0)	17	3	(17.6)	1.46	(0.46–4.62)	1.52	(0.48–4.81)
	Missing	73	13	(17.8)	1.47	(0.71–3.03)	1.82	(0.93–3.57)
**TB category**								
	New	113	13	(11.5)	ref		ref	
	Previously treated	175	26	(14.9)	1.29	(0.69–2.41)	1.22	(0.65–2.28)
	Missing	6	2	(33.3)	2.9	(0.84–10.03)	3.21	(1.04–9.92) *
**HIV**								
	Positive	3	2	(66.6)	4.82	(2.04–11.34)	7.14	(3.51–15.65) *
	Negative	253	35	(13.8)	ref		ref	
	Unknown	46	6	(13.0)	0.94	(0.42–2.11)	1.14	(0.52–2.51)
**Year of enrolment**								
	2016	99	20	(20.2)	1.76	(0.95–3.27)	1.79	(0.99–3.25)
	2017	72	8	(11.1)	0.97	(0.43–2.18)	0.90	(0.40–1.99)
	2018	131	15	(11.4)	ref			
**Culture**								
	Negative	61	6	(9.8)	ref			
	Positive	199	33	(16.6)	1.69	(0.74–3.83)	2.39	(1.09–5.24) *
	Unknown	42	4	(9.5)	0.97	(0.29–3.22)	1.44	(0.41–4.99)

#Row percentage.RR, risk ratio; aRR– adjusted risk ratio; CI, confident interval; BMI, body mass index; * statistically significant.
